# High intensity interval training exercise increases dopamine D2 levels and modulates brain dopamine signaling

**DOI:** 10.3389/fpubh.2023.1257629

**Published:** 2023-12-19

**Authors:** John Tyler, Madeline Podaras, Brittany Richardson, Nicole Roeder, Nikki Hammond, John Hamilton, Kenneth Blum, Mark Gold, David A. Baron, Panayotis K. Thanos

**Affiliations:** ^1^Behavioral Neuropharmacology and Neuroimaging Laboratory on Addictions (BNNLA), Research Institute on Addictions, Department of Pharmacology and Toxicology, Jacobs School of Medicine and Biomedical Sciences, University at Buffalo, Buffalo, NY, United States; ^2^Department of Biomedical Sciences, University at Buffalo, Buffalo, NY, United States; ^3^Department of Engineering and Applied Sciences, University at Buffalo, Buffalo, NY, United States; ^4^Department of Psychology, University at Buffalo, Buffalo, NY, United States; ^5^Center for Sports, Exercsie and Mental Health, Western University of Health Sciences, Pomona, CA, United States; ^6^Department of Psychiatry, Washington University School of Medicine, St. Louis, MO, United States

**Keywords:** autoradiography, addiction, dopamine, tyrosine, exercise, running, reward deficiency

## Abstract

**Background:**

Previous research has outlined the health benefits of exercise including its therapeutic potential for substance use disorders (SUD). These data have already been utilized and it is now common to find exercise as part of SUD treatment and relapse prevention programs. However, we need to better understand different exercise regimens and determine which would be the most beneficial for SUDs. Recently, high intensity interval training (HIIT) has gained attention in comparison with aerobic and resistance exercise. Little is known regarding the neurobiological mechanisms of HIIT, including its effects on dopamine signaling and receptor levels in the brain. The present study examined the effects of chronic HIIT exercise on dopamine signaling as measured by dopamine type 1-like receptor (D1R)-like, dopamine type 2-like receptor (D2R)-like, and tyrosine hydroxylase (TH) quantification in the brains of male and female rats as measured by [^3^H] SCH 23390 and [^3^H] spiperone autoradiography, and TH-immunoreactive optical density values.

**Methods:**

Rats were separated in two groups: sedentary and HIIT exercise. Exercise was on a treadmill for 30 min daily (10 3 min cycles) for six weeks with progressive speed increased up to 0.8 mph (21.5 m/min).

**Results:**

Results showed for D2R-like binding, a significant effect across the ventral caudate putamen (V CPU) between sexes, such that mean D2R-like binding was 14% greater for males than females. In the nucleus accumbens shell (Nac Shell), the HIIT Exercise rats showed 16% greater D2R-like binding as compared to the sedentary rats. No significant effects of HIIT exercise were found across groups for brain D1R-like binding levels or TH expression.

**Conclusion:**

These results suggest that HIIT exercise can modulate dopamine signaling by way of increased D2R. These findings support the premise that HIIT exercise plays an important role in dopamine signaling and, may provide a potential mechanism for how HIIT exercise can impact the brain and behavior.

## Introduction

The therapeutic potential of exercise in medicine continues to grow as the application of its benefits is realized (1). Exercise has been shown to reduce the risk of several diseases, and improve overall health (2–4). Exercise intervention has also shown particular efficacy in risk reduction and treatment of substance use disorders (SUDs) (5–11).

In the US there has been a significant increase in substance abuse in the past 5 years, with 40.3 million individuals currently affected, coupled with a $6.8 billion increase in federal spending for drug control from 2015 to 2020 (12). Considering current trends, exercise is a valuable intervention given its cost-effectiveness, accessibility, and ease of use. Clinical trials have proven the efficacy of exercise in treating SUDs while animal models identified its effectiveness in preventing initiation, escalation, and relapse. Exercise is a valuable treatment modality for SUDs as it positively modulates dopamine pathways, specifically the mesolimbic reward pathway, back to a healthy baseline. Given the efficacy of exercise as a therapeutic adjunct for SUDs, research continues with the goal of better understanding the exercise regimen and architecture for maximizing effects and benefits.

As exercise research evolves, more evidence continues to support the potential of high intensity interval training (HIIT) in both preclinical and clinical studies. HIIT is defined by the American College of Sports Medicine as short bouts of high intensity, greater than 65% of maximal capacity, anaerobic exercise “alternating with very short bouts of less intense recovery” (13). In stark contrast, moderate intensity aerobic exercise (MIAE) consists of intensities ranging from 40% to 60% with little fluctuation during exercise (14). HIIT has proven an effective exercise regimen in general given its greater improvements in VO_2_ max compared to MIAE (15), significant enhancement of working memory capacity and cognitive performance (16), and marked reduction of fasting glucose levels and insulin resistance (17). More important to its application in the treatment of addiction is its outstanding efficacy in the field. HIIT is preferred over MIAE by inactive individuals and is shown to have higher rates of adherence to the regime over time. Moreover, HIIT may possess greater reinforcing properties allowing for better reception and retention among sedentary individuals (18). This increased adherence is especially important given the major difficulties experienced by drug addicts in adhering to certain exercise types (19). HIIT, compared to vigorous-intensity continuous training, was shown to produce higher serum neurotrophin-3 concentrations in humans which plays an important role in the attenuation of craving and reward dependence through a preponderance of dopaminergic regulation (20). When comparing cortisol levels among different endurance exercise protocols, levels were significantly higher with the HIIT protocol (21). Increased cortisol levels following exercise were associated with decreased substance craving. Significantly higher insulin-like growth factor1 (IGF-1) levels were also identified for participants in a HIIT group versus the control resulting in increased hippocampal BDNF expression and neuronal differentiation (22). HIIT provides greater benefits for physical and mental health compared to MIAE (23, 24). Furthermore, patients with SUDs have displayed significant improvements on depression, anxiety, and substance craving scales following a HIIT regimen compared to the control group (22). Other research has confirmed these findings and highlighted additional health benefits conferred by HIIT in those with SUDs such as decreased cardiovascular disease and premature death risk (25).

Exercise’s efficacy in treating addiction is largely linked to its ability to positively modulate the mesolimbic reward pathway. The mesolimbic pathway transports dopamine from the ventral tegmental area (VTA) to the nucleus accumbens and amygdala. The nucleus accumbens is located within the striatum and is of particular importance to addiction as it is believed to modulate reward, desire, and the placebo effect (26). Previous research reported reductions in dopamine D1R-like binding as a neurobiological factor contributing to exercise-induced attenuation of drug-seeking behavior (6–8); however, dopaminergic signaling in response to HIIT exercise has yet to be elucidated. Past studies have shown that chronic treadmill exercise resulted in lower levels of D1R-like binding in the ventral striatum and higher D2R-like binding in several subregions of the dorsal striatum (6–8). Blocking dopamine D1 receptors in the nucleus accumbens core and shell attenuated ethanol-seeking behavior in rats (27). Dopamine D1 receptor knock-out mice were also shown to self-administer significantly less cocaine than wild type (28). This pathway is further supported by previous findings that antagonists of D1-like receptors facilitated drug extinction and attenuated drug-seeking behavior (29). These findings suggest that reductions in D1R-like binding contribute to the attenuation of drug-seeking behavior, thus supporting the efficacy of exercise in treating addiction due to its ability to reduce D1R-like binding.

Conversly, increasing D1R-like binding is linked to exacerbation of addiction symptomology. Previous research has shown that increases in D1R and a decreases in D2R are linked to enhanced compulsive and drug-seeking behavior (30). D1R stimulation has proven important for drug reward and conditioned association (30). This was further supported by postmortem studies of methamphetamine users which revealed significant increases in D1R in the NAc (31). Considering current research, it can be reasonably suggested that increases in D1R exacerbate addiction while reductions in D1R may help alleviate addiction symptomology, specifically drug-seeking behavior.

Previous research has also highlighted exercise’s ability to increase dopamine type 2-like receptor levels and to attenuate drug and alcohol consumption in rats in a sex-dependent (8). These results suggest that an increase in dopamine type 2-like receptor levels may be causally related to attenuation of drug and alcohol consumption (32–37). Robison et al. (8, 11) highlighted how exercise’s benefits on the dopamine (DA) system may be dependent on the intensity or “dose” of the exercise itself; which would impact the reward pathway differently. In mouse models of Parkinson’s disease, high-intensity exercise was shown to increase striatal dopamine D2R expression (38). Additional studies on treadmill running have confirmed the D2R enhancement conferred by exercise (39–41). The increase of D2R-like binding as a result of exercise has also been suggested to attenuate aversion to pain and act as an analgesia (42). In this study, the D2R antagonist was only able to attenuate the pain-blocking response in the presence of the stressor of exercise. Particularly, HIIT would be a high-intensity, short-duration stressor that is suggested to provide a significant analgesic effect. In contrast, chronic pain decreases D2R which may have implications for drug-seeking behavior, suggesting a possible chemical connection between pain medication reliance and addiction (43). Increased D2R-like binding has also been shown to have implications for extending lifespan through the GOA-1-DGK-1-PKC/PKD signaling pathway (44). The dopamine receptor D2 gene (DRD2) is known to play an important role in determining the effectiveness of exercise through the modulation of lipid and carbohydrate metabolism (45). Exercise has also been shown to increase D2R-like binding promoting defensive behavior and conditioned place avoidance (46). Decreased D2 receptor levels, specifically in the striatum, have been associated with increased addictive behavior and increased impulsivity, while increased D2 receptor signaling is associated with increased motivation for recovery from addiction (47). Polymorphisms that elicit reduced D2R densities and binding affinity are significantly associated with nicotine, alcohol, heroin, and cocaine dependence (48–51). Perhaps one of the most replicated findings, however, is the association of cocaine abuse and alcohol dependence with significantly decreased D2R striatal availability (47). Preclinical research has linked increased D2 receptor signaling with enhanced motivation and decreased D2 receptor signaling with attenuated motivation (52). Additionally, successful treatment of depression is correlated with increased striatal D2R-like binding (53). This is important as depressive symptoms including suicide ideation, are known to play a key role in impeding remission efforts, whereby the top candidate gene was the D2R using large GWAS (54, 55).

Tyrosine hydroxylase (TH) levels are another important neurobiological marker of dopamine signaling. TH is the rate-limiting enzyme in the synthesis of catecholamine neurotransmitters, notably dopamine; therefore, TH levels effectively correlate with dopamine bioavailability (56, 57). Past research has indicated that drugs of abuse attenuate tyrosine hydroxylase activity through methylation of the tyrosine hydroxylase gene effectively decreasing TH levels (58, 59). Multiple differing models have highlighted elevations in tyrosine hydroxylase levels conferred by exercise (60–63). Therefore, these studies suggest that exercise modulates TH in such a way that may have therapeutic potential for SUD and, thus, TH levels are important to being examined.

The present study examined the effect of HIIT treadmill exercise on dopamine signaling as measured by D1R, D2R, and TH levels in the brains of rats as compared to sedentary rats. These data will aid our understanding of how varying intensities of prescribed exercise can impact dopamine signaling and this information would be important in exercise neuroscience and medicine.

## Methods

### Subjects

Male (*n* = 15) and female (*n* = 16) Lewis rats (Charles River) at 3 weeks of age were individually housed under standard laboratory conditions at 22.0-C T 2-C with a 12 h reverse light/dark cycle (lights off: 06:00 AM to 06:00 PM). Food and water were available *ad libitum* for the duration of the study. All subjects were handled daily. The experiment was conducted in accordance with the National Academy of Sciences Guide for the Care and Use of Laboratory Animals (1996) and University at Buffalo Institutional Animal Care and Use Committee.

### Treadmill

A custom-made motorized treadmill divided into eight lanes by Plexiglas walls and by a sheet of metal at its end to keep the rats enclosed on the treadmill was used, as previosuly described (8). The dimensions of the running lanes were 56 cm long, 9 cm wide, and 31 cm high.

### Exercise regimen

The rats received a 7 days acclimation period upon arrival. Groups were randomly assigned. *n* = 8 male rats and *n* = 8 female rats were placed in the HIIT group while *n* = 7 male rats and *n* = 8 female rats were selected for the sedentary group. On the first day following the acclimation period, animals began their exercise regimen of 6 weeks. The rats within the HIIT cohort began at 10 m/min for 30 min. This regimen consisted of 3 min intervals in which the rats ran for 2 min followed by a 1 min break and was repeated 10 times for a total of 30 min. After 5 days, the speed was increased by increments of 2.87 m/min each day until a speed of 21.46 m/min was reached to allow for steady acclimation to the exercise regimen. This speed was chosen as it essentially doubles previous MIAE speeds of 10 m/min to significantly increase intensity to greater than 65% maximal capacity (8). The exercise continued at the top speed for the remainder of the exercise regimen. The HIIT regimen ran 7 days a week. The animals were monitored for the entire span of their exercise regimen. All exercise sessions were performed during the subjects’ dark cycle (06:00 AM to 06:00 PM). Sedentary rats were exposed to the same environment and hadling but without treadmill being on.

### D1 autoradiography

Dopamine type 1-like receptor (D1R) expression was assessed using [^3^H] SCH 23390 autoradiography. Binding was performed as previously described (8). Briefly, slides were preincubated for 60 min at room temperature in 50 mM Tris HCl buffer (120 mM NaCl, 5 mM KCl, 2 mM CaCl_2_, 1 mM MgCl_2_, pH = 7.4). Next, 2.5 nM [^3^H] SCH 23390 (specific activity = 85 Ci/mmol) and 40 nM ketanserin were added to the pre-incubation buffer followed by an additional 60 min incubation at room temperature. Non-specific binding was determined in the presence of 1 μM flupenthixol. Brain section slides were then washed 2× 5 min at 4°C in pre-incubation buffer followed by brief immersion at 4°C in dH_2_O.

### D2 autoradiography

Dopamine type 2-like receptor (D2R) expression was assessed using [^3^H] Spiperone autoradiography. Binding was performed as previously described (8). Briefly, slides were preincubated for 60 min at room temperature in 50 mM Tris 7 HCl buffer (120 mM NaCl, 5 mM KCl, 2 mM CaCl_2_, 1 mM MgCl_2_, pH = 7.4). Next, 0.5 nM [^3^H] Spiperone (specific activity = 16.2 Ci/mmol) and 40 nM ketanserin were added to the pre-incubation buffer followed by an additional 60 min incubation at room temperature. Non-specific binding was determined in the presence of 10 μM sulpride. Brain section slides were then washed 2× 5 min at 4°C in pre-incubation buffer followed by brief immersion at 4°C in dH_2_O.

### TH immunohistochemistry

Brain section slides were initially dehydrated in 90% ethanol for 10 min at room temperature (64). TH IHC was performed as previously described but with a few modifications (65). Next, 3× 5 min washes were completed in 1X PBS. Slides were then blocked with a solution containing 0.4% Trition-X, 10% normal goat serum, and 1% H_2_O_2_ for 30 min at room temperature followed by 24 h incubation at 4°C with TH primary antibody: Rabbit anti-TH antibody (1:2000, Thermofischer). After five washes in 1X PBS with 0.4% Triton-X (PBS-T), slides were sequentially incubated in biotinylated goat anti-rabbit (1:800, Vector Laboratories, Burlingame, CA) for 1 h and avidin-biotin complex (ABC Kits; Vector Laboratories, Burlingame, CA) for 45 min separated by five washes in PBS-T. Following five washes in PBS-T, immunostaining was visualized using 3,3-diaminobensidine (Vector, Burlingame, CA), mixed with H_2_O_2_. Slides were then washed 4× 5 min in PBS-T, dipped into dH_2_O, and coverslipped with Permount mounting medium (Fisher Scientific). After TH-staining, sections from the striatum were imaged with a digital camera connected to a compound microscope (National DC3-163 Digital Microscope) and with a computer equipped with MOTIC imaging software. Density in the striatum was measured using ImageJ software.

### Regions of interest

Bound slides and tritium standards on glass slides were opposed to Kodak MR Film. The film was scanned at 1,200 dpi, and images were quantified using ImageJ software. The regions of interest include the nucleus accumbens shell (NAc Shell), nucleus accumbens core (NAc Core), ventromedial caudate putamen (VM CPU), ventrolateral caudate putamen (VL CPU), dorsolateral caudate putamen (DL CPU), dorsomedial caudate putamen (DM CPU), olfactory tubercle (OT), and substantia nigra (SNR) were analyzed for [^3^H]SCH 23390 and [^3^H] Spiperone binding and TH Immunohistochemistry.

### Statistical analysis

**Figure 1:**
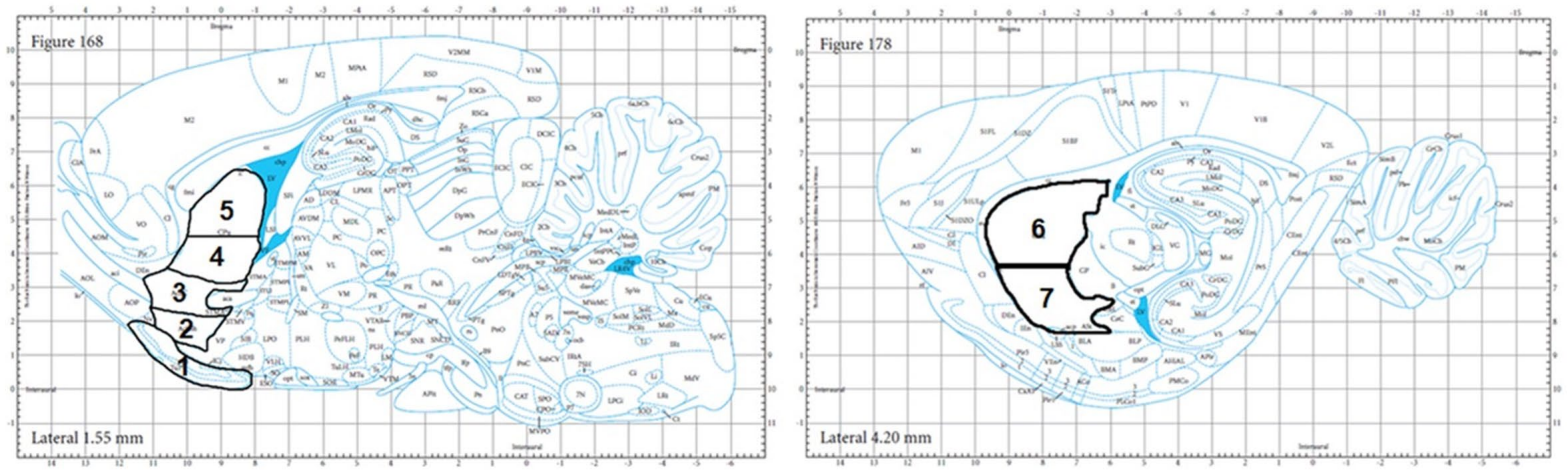
Brain anatomical atlas images of the regions analyzed: the OT (#1), NAC Shell (#2), NAC Core (#3), VM CPU (#4), DM CPU (#5), DL CPU (#6), and VL CPU (#7) as adopted from the Paxinos & Watson rat brain atlas.

For each region of interest, two-way ANOVA was conducted to determine exercise or sex differences with a dependent variable of D1R, D2R, or TH binding. The significance level was set at *α* = 0.05 and all statistical analyses were performed with Prism9 Graphpad software version 9.4.1. Post-hoc tests were performed on significant main effects within the region of interest using Sidak’s multiple comparison test (see Figure 1).

## Results

### [^3^H]SCH 23390 autoradiography

**Figure 2:**
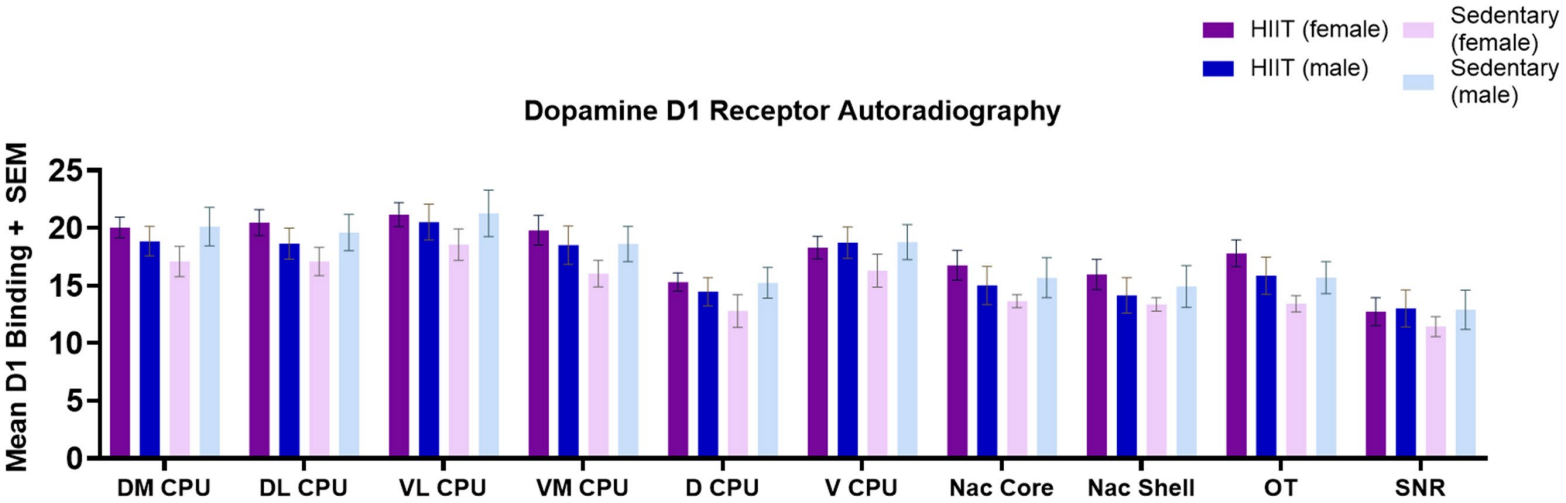
Quantitative autoradiography of [^3^H]SCH 23390 (D1R-like) binding levels within the nucleus accumbens shell (Nac Shell), nucleus accumbens core (Nac Core), ventromedial caudate putamen (VL CPU), ventrolateral caudate putamen (VL CPU), dorsolateral caudate putamen (DL CPU), dorsomedial caudate putamen (DM CPU), dorsal caudate putamen (D CPU), ventral caudate putamen (V CPU), olfactory tubercle (OT), and substantia nigra (SNR) across exercise treatment groups. Measurements of the following regions were carried out at the bregma coordinates taken from the Paxinos & Watson rat brain atlas. Values are expressed as total [^3^H]SCH 23390 binding means ± S.E.M. for D1R receptors. No significant group differences were observed (*p* > 0.05). Each bar represents the group mean for dopamine D1 receptor binding.

No significant effects of HIIT exercise were found across groups for brain [^3^H]SCH 23390 binding levels (D1R-like). Specifically, in the DM CPU [*F*(1,25) = 2.65; *p* > 0.05], DL CPU [*F*(1,25) = 2.60; *p* > 0.05], VL CPU [*F*(1,25) = 0.48; *p* > 0.05], VM CPU [*F*(1,25) = 1.81; *p* > 0.05], D CPU [*F*(1,25) = 1.74; *p* > 0.05], V CPU [*F*(1,25) = 0.57; *p* > 0.05], Nac Core [*F*(1,25) = 1.92; *p* > 0.05], Nac Shell [*F*(1,25) = 1.59; *p* > 0.05], OT [*F*(1,25) = 2.93; *p* > 0.05], and SNR [*F*(1,25) = 0.19; *p* > 0.05]. Two-way ANOVA found no significant interactions or main effects across groups (*p* > 0.05; Figure 2). Across D1R-like binding, a general trend towards significance was observed for HIIT females compared to the sedentary female group with the strongest trend towards significance observed in the OT [*F*(1,25) = 2.93; *p* < 0.1]. This trend reflected an increase in D1R-like binding in all regions of interest for the HIIT female rats compared with the sedentary female rats.

### [^3^H] Spiperone autoradiography

**Figure 3:**
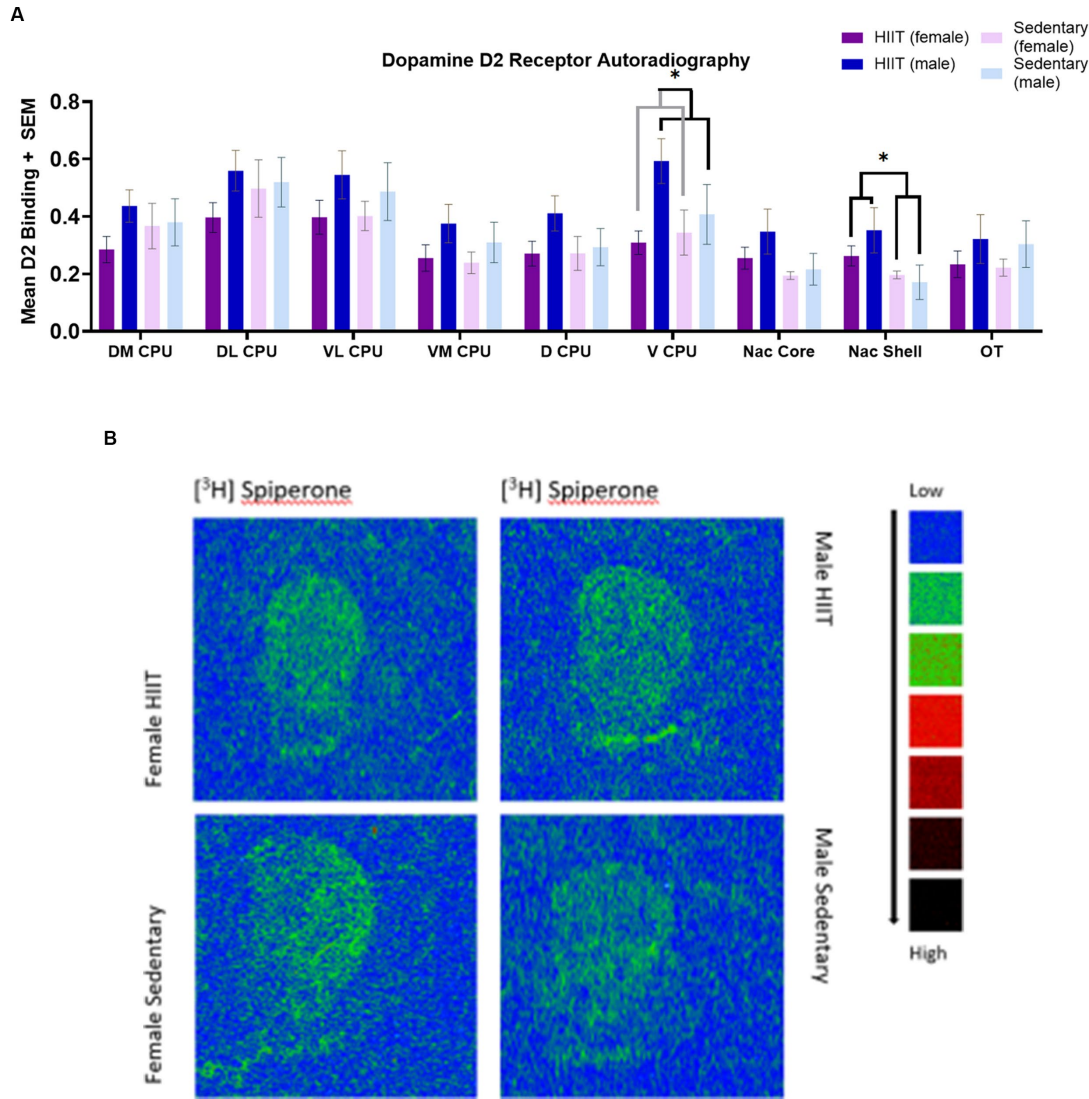
**(A)** Quantitative autoradiography of [^3^H] Spiperone (D2R-like) binding levels within the nucleus accumbens shell (Nac Shell), nucleus accumbens core (Nac core), ventromedial caudate putamen (VL CPU), ventrolateral caudate putamen (VL CPU), dorsolateral caudate putamen (DL CPU), dorsomedial caudate putamen (DM CPU), dorsal caudate putamen (D CPU), ventral caudate putamen (VCPU), and olfactory tubercle (OT) across exercise treatment groups. Measurements of the following regions were carried out at the bregma coordinates taken from the Paxinos & Watson rat brain atlas. Values are expressed as total [^3^H] Spiperone binding means ± S.E.M. for D2R receptors. Two-way ANOVA found a main effect across the ventral caudate putamen (V CPU) for sex [*F*(1,26) = 4.84; *p* < 0.04], such that D2R-like binding was significantly greater for males than females. Across the nucleus accumbens shell (Nac Shell), a main effect was found for HIIT Exercise [*F*(1,26) = 5.27; *p* < 0.03], such that mean D2R-like binding was significantly greater for the HIIT group than the sedentary group. Each bar represents the group mean for dopamine D2 receptor binding. **(B)** A representative image of D2 autoradiographic binding distribution Specific images were taken from [^3^H] Spiperone binding in the rat brain. Representative images were taken at approximately bregma level +1.32 mm.

Two-way ANOVA found a main effect across the ventral caudate putamen (V CPU) for sex [*F*(1,26) = 4.84; *p* < 0.04; Figures 3A,B], such that mean [^3^H] spiperone (D2R-like) binding was significantly greater for males than females. The main effect of HIIT Exercise [*F*(1,26) = 0.91; *p* > 0.05] and the HIIT-sex interaction [*F*(1,26) = 1.96; *p* > 0.05] were not significant in the V CPU.

In the nucleus accumbens shell (Nac Shell), a main effect was found for HIIT Exercise [*F*(1,26) = 5.27; *p* < 0.03], such that mean D2R-like binding was significantly greater for the HIIT group than the sedentary group. The main effect of sex [*F*(1,26) = 0.36; *p* > 0.05] and the HIIT-sex interaction [*F*(1,26) = 1.14; *p* > 0.05] were not significant in the Nac Shell. No other main effects were observed in the Nac Shell (*p* > 0.05; Figures 3A,B).

No significant effects were reported across the DM CPU [*F*(1,26) = 1.94; *p* > 0.05], DL CPU [*F*(1,26) = 0.76; *p* > 0.05], VL CPU [*F*(1,26) = 0.17; *p* > 0.05], VM CPU [*F*(1,26) = 0.19; *p* > 0.05], D CPU [*F*(1,26) = 1.03; *p* > 0.05], Nac Core [*F*(1,26) = 0.44; *p* > 0.05], and OT [*F*(1,26) = 0.0021; *p* > 0.05].

### TH immunohistochemistry

**Figure 4:**
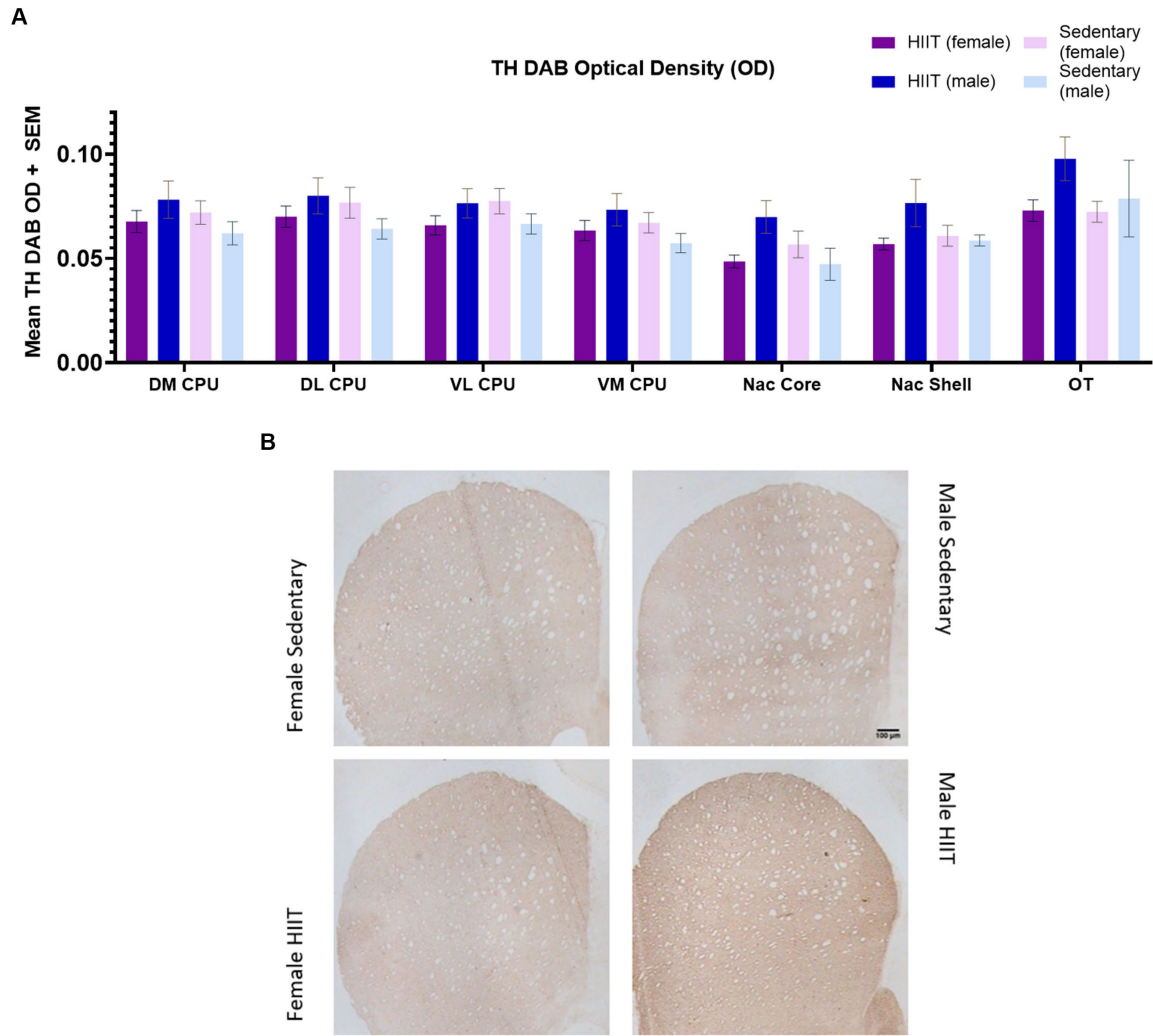
**(A)** Two-way ANOVA found no significant interactions or main effects across groups (*p* > 0.05). Each bar represents the group mean for TH DAB optical density. **(B)** Effect of treadmill exercise on tyrosine hydroxylase (TH)-immunoreactive fibers in the striatum of the Lewis rats. Photomicrographs of TH-positive fibers in the striatum. The scale bar represents 100 μm.

No significant effects of HIIT exercise were found across groups for TH-immunoreactive fiber densities across the DM CPU [*F*(1,20) = 2.03; *p* > 0.05], DL CPU [*F*(1,20) = 2.24; *p* > 0.05], VL CPU [*F*(1,20) = 2.93; *p* > 0.05], VM CPU [*F*(1,20) = 2.44; *p* > 0.05], Nac Core [*F*(1,20) = 4.24; *p* > 0.05], Nac Shell [*F*(1,20) = 1.98; *p* > 0.05], and OT [*F*(1,20) = 0.99; *p* > 0.05; Figures 4A,B].

## Discussion

The current study examined the effects of chronic HIIT exercise on dopamine (DA) signaling in rats. Results demonstrated that chronic HIIT exercise produced significant changes in D2R binding levels in the Nac Shell. Specifically, the mean D2R-like binding was 16% greater in the HIIT rats as compared to the sedentary rats. Previous research has reported an increase in D2R after a moderate-intensity treadmill exercise regimen which was shown to attenuate drug-seeking behaviors (8). Sex-dependent differences were mainly observed in the V CPU for D2R-like binding wherein binding was 14% greater for males than females. This finding suggests sex-dependent differences in D2R-binding levels.

For D2R-like binding, we observed an effect of HIIT exercise in the Nac Shell. This brain region is of particular importance due to its critical role in the mesolimbic pathway and association with the modulation of reward and desire. The mesolimbic pathway is highly influenced by dopamine signaling including D1 and D2 receptor levels (66). Past research found that steady moderate treadmill exercise elicited an increase in D2R-like binding in several subregions of the dorsal and ventral striatum, specifically within the DM CPU, VL CPU, VM CPU, and OT (8). The CPU is believed to control compulsive aspects of addiction, such as drug-seeking behavior (67). Previous findings outlined how treadmill exercise enhanced D2R in the basal ganglia, a brain region responsible for SUD habit formation through control of reward (38–40). Additionally, environmental enrichment with wheel running has also been associated with a significant increase in D2R gene expression (68). Clinical research has confirmed this association between D2R-like binding and exercise in which higher-intensity physical activity was associated with greater D_2/3_ receptor availability (69). Our study’s findings of the increased Nac Shell D2R-like binding agree with these aforementioned studies.

Thus, we may speculate that HIIT also attenuates drug-seeking behavior through its modulation of D2R binding. The present study, along with many others, have confirmed the increase in D2R conferred by exercise, including HIIT exercise (8, 11, 38–41). Rodent models have specifically outlined a causal link between exercise-induced D2R enhancement and attenuation of drug consumption (6–8). This exercise-induced enhancement of D2R is also linked to conditoned place avoidance, enhanced motivation, and decreased depressive symptoms (46, 52, 53). These are important factors known to play key roles in remission efforts. Furthermore, enhancement of D2R signaling is associated with increased motiviation for recovery from addiction, while reduction in D2R levels in the striatum is associated with increased impulsivity and addictive behavior (47). Since previous research suggests that positive modulation of dopamine is likely dependent on exercise intensity, we may speculate that exercise remains a beneficial treatment modality for addiction due to its enhancement of D2R and that HIIT may provide exceptional efficacy due to its greater intensity.

In contrast to D2R, HIIT exercise did not to have a significant effect on D1R-like binding, across all regions of interest in both male and female rats. Previous research using a steady and moderate-intensity treadmill exercise regimen—showed a reduction of D1R (8). Compared to moderate-intensity aerobic continuous exercise (MI-ACE), our findings point to neurochemical differences in D1R levels between a HIIT exercise regimen and a MI-ACE regimen.

TH levels are known to correlate with dopamine bioavailability and effectively predict dopamine levels (56, 57). As indicated by previous research, TH activity decreases with the loss of dopamine neurons as TH is the rate-limiting enzyme in the synthesis of dopamine (70). The present study showed that chronic HIIT exercise did not produce significant changes in TH levels, thus suggesting insignificant changes in DA levels.

Insignificant modulation of TH and D1R may be explained by a suggested upper limit to neurotrophic factors. Previous studies have indicated that exercise-induced modulation of neurotrophic factors is occasionally absent in healthy controls, despite observing significant effects of exercise in animal models representing a diseased condition subject to the same protocol (63). This phenomenon may be explained by a sort of natural ceiling or upper limit to neurotrophic modulation. The healthy rats are already at homeostasis and thus much closer to an upper limit than rats of a diseased model such as Parkinson’s disease or SUDs where baseline dopamine signaling is already significantly imparied. Therefore, the healthy rats may not show a significant effect when the diseased model does because the diseased model has a much larger increase in neurotrophic factor from deficit to upper limit rather than from homeostasis to upper limit. Likewise, the rodents utlized in our study were healthy and thus theoretically had a less significant range baseline to upper limit than would a diseased or addicted model. Hence, it would be unreasonable to assume that an addicted model would not exhibit positive modulation of TH and D1R with HIIT solely based on the insignificant effects observed in our healthy model. This topic warrants further exploration and comparison between addicted and healthy models of exercise-induced dopamine signaling.

The disparate findings in D1R binding demonstrated by HIIT exercise compared to moderate-intensity exercise could be explained by exercise intensity. Chronic high-level voluntary wheel running has been shown to produce lower levels of D2R mRNA and higher levels of ΔFosB/FosB immunoreactivity and TH mRNA in the NAc Core, NAc, and the ventral tegmental area (VTA) respectively, exhibiting a resemblance to chronic drug exposure (71, 72). Stimulation of D1-expressing medium spiny neurons has been shown to enhance sensitivity to drugs of abuse (26, 73–75). Additionally, chronic methamphetamine users are known to exhibit significantly higher D1R levels (31). Previous research has outlined D1R stimulation as the primary mechanism for drug reward and conditioned association (30). The results of the present study suggest that HIIT exercise regimen resulted in increased D2R levels in the brain. Therefore, it would be beneficial for future studies to directly examine various intensities of exercise and their potential effects on substance abuse behavior (76).

A plausible contributor to varied responses of exercise on D1R, D2R and TH expression can be overtraining; however, this notion was refuted for our study. A recent study on HIIT analyzed the threshold for overtraining in humans (77). Current HIIT training recommendations advise that HIIT be followed by low-to-moderate intensity training for at least the first 48 h to ensure proper recovery, especially in untrained individuals (13, 77). Furthermore, HIIT guidelines also recommend 1–2 recovery days per week and no more than a weekly increase of 10% in training time or intensity (78). Overtraining has been shown on numerous accounts to induce neurotransmitter and enzyme disturbances including disturbances of dopamine and modulation of TH expression (60–63, 79, 80). Furthermore, overtraining can dampen and even reverse some of the positive effects of exercise; therefore, it is imperative to screen for signs and symptoms of overtraining syndrome to validate related findings.

Our present study utilized a HIIT exercise regimen consisting of daily 30 min training sessions amounting to a total of 210 min of HIIT per week for 6 weeks. Although a HIIT overtraining threshold has yet to be identified for rodents, corticosterone ELISA analysis found no significant difference in corticosterone levels between HIIT treated rats relative to sedentary rats (72). Additionally, a significant decrease in animal body weight, which is an accepted sign of overtraining, was not observed throughout this HIIT regimen (81, 82). These findings indicate that our HIIT regimen did not induce overtraining syndrome in the exercise group thus validating our findings.

The varied effects of our HIIT results compared to other exercise routines point to the importance of exercise specificity in clinical application. It is evident that different exercise regimens are better at producing certain results and treating specific conditions. Resistance training is a prime example with its unmatched efficacy in treating osteoporosis and sarcopenia (83). Other research suggests that MIAE may be the best regimen for reducing all-cause mortality and that high-dose resistance training may be the worst followed by vigorous exercise possibly due to increased vessel calcification (84, 85). These differences are likely transferable to treating addiction; wherein, there is a most effective and least effective or possibly even counterproductive exercise regimen in treating addiction. The present study findings support further research to elucidate exercise regimen or exercise perscription differences and their efficacy in therapeutic tools combating many psychiatric disorders, including substance use disorders.

**Figure 5:**
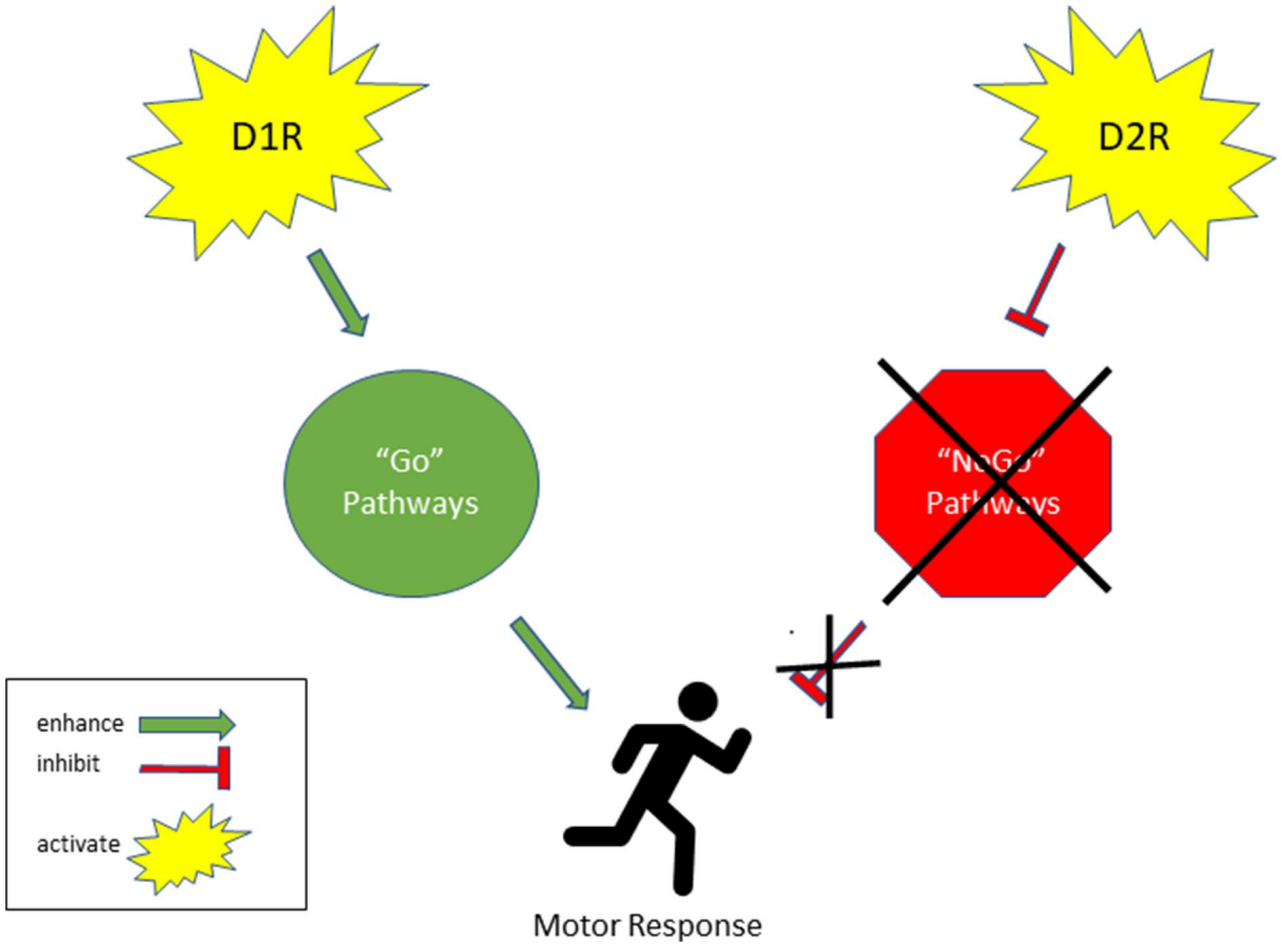
Effect of D1R and D2R activation on motor responses.

The results of this study alluded to sex-dependent differences in dopamine signaling. As discussed, past studies have shown that female rats are more susceptible to addiction (86–88). These sex-dependent effects were observed in our D2R data. The male rats showed 14% greater D2R binding levels in the V CPU as compared to females. The greater levels of D2R indicate sex-dependent differences in D2R binding. Striatal D1 receptors enhance the synaptic activity of “Go” pathways, promoting the likelihood of triggering a specific response. Conversely, striatal D2 receptor activation inhibits “NoGo” pathways, which in turn increases the probability of initiating a particular response (89). Moreover, D1-expressing neurons directly activate motor responses, while D2-expressing neurons indirectly trigger motor responses by removing inhibitory signals (Figure 5). This data alludes to the notion that there are sex differences in how exercise may mediate neurochemistry and thus a sex difference in their utility and therapeutic potential. Careful consideration is needed in terms of exercise prescription. Furthermore, exercise prescription may be further influenced by sex and so further research is warranted on this. Since our study focused on the neurological effects of exercise, future studies should explore how HIIT exercise affects drug-seeking and even non-drug seeking behaviors like overeating and gambling. This may provide insight into the best exercise-based treatment for drug-use attenuation.

## Conclusion

SUD programs have had considerable interest in dopamine as relative dopamine deficiences have been considered central to the pathophysiology of human addiction, drugs of abuse-related dysphoria, and craving (90, 91). The present findings showed that daily HIIT treadmill exercise had a significant effect on D2R-like binding in the Nac Shell, as the exercise group displayed 16% higher D2R-like binding compared to the sedentary group. Sex differences were also observed in the V CPU wherein males showed 14% greater D2R-like binding than females. HIIT exercise did not impact D1R-like binding or TH expression as compared to a sedentary group.

## Data availability statement

The raw data supporting the conclusions of this article will be made available by the authors, without undue reservation.

## Ethics statement

The animal study was approved by the Institutional Animal Care and Use Committee (IACUC). The study was conducted in accordance with the local legislation and institutional requirements.

## Author contributions

JT: Data curation, Formal analysis, Funding acquisition, Investigation, Methodology, Project administration, Resources, Software, Supervision, Validation, Visualization, Writing – original draft, Writing – review & editing. MP: Data curation, Formal analysis, Investigation, Methodology, Writing – original draft, Writing – review & editing. BR: Formal analysis, Investigation, Methodology, Supervision, Writing – review & editing. NR: Formal analysis, Investigation, Methodology, Supervision, Writing – review & editing. NH: Formal analysis, Investigation, Methodology, Supervision, Writing – review & editing. JH: Formal analysis, Investigation, Methodology, Supervision, Writing – review & editing. KB: Writing – review & editing. MG: Funding acquisition, Writing – review & editing. DB: Writing – review & editing. PT: Conceptualization, Data curation, Formal analysis, Funding acquisition, Investigation, Methodology, Project administration, Resources, Software, Supervision, Validation, Visualization, Writing – original draft, Writing – review & editing.
